# Prevalence of nontuberculous mycobacteria and the emergence of rare species in Henan Province, China

**DOI:** 10.1017/S095026882400061X

**Published:** 2024-05-06

**Authors:** Shi Hongmei, Liang Ruixia, Li Jiankang, Wu Han, Zhao Chang, Mo Yan Yan, Wang Zi Kang, Li Fu Li, Ding Cheng Zhi, Liu Xin

**Affiliations:** Clinical Laboratory, Tuberculosis Department, Henan Chest Hospital, Zhengzhou, China

**Keywords:** mycobacteria, infectious disease epidemiology, nontuberculous mycobacteria, Prevalence, *M. intracellulare*

## Abstract

Nontuberculous mycobacteria (NTM) is a large group of mycobacteria other than the *Mycobacterium tuberculosis* complex and *Mycobacterium leprae.* Epidemiological investigations have found that the incidence of NTM infections is increasing in China, and it is naturally resistant to many antibiotics. Therefore, studies of NTM species in clinical isolates are useful for understanding the epidemiology of NTM infections. The present study aimed to investigate the incidence of NTM infections and types of NTM species. Of the 420 samples collected, 285 were positive for *M. tuberculosis*, 62 samples were negative, and the remaining 73 samples contained NTM, including 35 (8.3%) only NTM and 38 (9%) mixed (*M. tuberculosis* and NTM). The most prevalent NTM species were *Mycobacterium intracellulare* (30.1%), followed by *Mycobacterium abscessus* (15%) and *M. triviale* (12%). *M. gordonae* infection was detected in 9.5% of total NTM-positive cases. Moreover, this study reports the presence of *Mycobacterium nonchromogenicum* infection and a high prevalence of *M. triviale* for the first time in Henan. *M. intracellulare* is the most prevalent, accompanied by some emerging NTM species, including *M. nonchromogenicum* and a high prevalence of *M. triviale* in Henan Province. Monitoring NTM transmission and epidemiology could enhance mycobacteriosis management in future.

## Introduction

Nontuberculous mycobacteria (NTM) is a large group of mycobacteria other than the *Mycobacterium tuberculosis* complex (MTBC) and *Mycobacterium leprae.* In recent years, the isolation rate of NTM has been increasing annually, attracting the attention of healthcare management and researchers [1]. So far, over 200 species and 19 subspecies of NTM have been identified [2]. Recent data showed an upward trajectory in pulmonary NTM infections (82.1%) and disease (66.7%). The overall annual rate of change for NTM infections and disease per 100,000 persons/year was 4.0% and 4.1%, respectively. The absolute numbers of NTM infections and disease showed an overall annual change of 2.0 and 0.5, respectively. Infections and disease caused by the *Mycobacterium avium* complex and infections caused by the *Mycobacterium abscessus* complex (MABC) showed increasing trends [3].

Like many other countries, China faces the emerging challenges of NTM infections due to the increasing prevalence of conditions such as chronic obstructive pulmonary disease (COPD) and the growing population of elderly people and immunocompromised patients. These conditions may contribute to a higher risk of NTM infections. Additionally, advancements in diagnostic techniques, including Fujifilm SILVAMP (FujiLAM; Japan) for TB LAM test to rapidly detect *M. tuberculosis* [4], and increased awareness among healthcare professionals might lead to improved detection and reporting of NTM infections, including *M. triviale* infections.

According to a nationwide multicentre study published in 2021, *Mycobacterium intracellulare* (52.6%) had the highest isolation rate among NTM in China, followed by the MABC (23.1%) [5]. In Guangzhou, China, the isolation rate of NTM is higher than the national average, with *M. avium*–*M. intracellulare* complex (MAC, 44.5%), MABC (40.5%), and *Mycobacterium kansasii* (10.0%) being the most prevalent [6].

The emergence of NTM infections may further complicate tuberculosis (TB) treatment and diagnosis in the near future [3]. Recently (data not published), many NTM have also been observed in health centres. However, species and subspecies identification data are unavailable in some regions, including Nanyang. Pulmonary NTM infections primarily manifest as chronic lung diseases that resemble TB, with symptoms like cough, fatigue, weight loss, and difficulty breathing [7]. Extrapulmonary NTM infections can affect various body parts, including skin, lymph nodes, joints, and bones, causing a wide spectrum of symptoms depending on the site of infection [8].

The diagnosis of NTM infections is mainly based on clinical, radiological, and microbiological results [9]. The clinical course of NTM pulmonary infections is heterogeneous, and the disease is associated with considerable mortality and morbidity [10]. Treatment decisions for NTM infections depend on identifying isolated species, drug sensitivity testing, and disease severity [10]. However, identifying NTM species is not performed in some regions, which may lead to its transmission to the population. NTM are abundant in soil and water, which may be transmitted from animals to humans [11]. In Thailand and Vietnam, NTM constitute 21% of mycobacterial infections, and those who develop lung disease are at high risk of developing NTM infections. In a previous study, of a sample of 218 patients, 30% had potential NTM pulmonary disease, 4% were confirmed, and 5% were diagnosed with disseminated NTM disease. Patients with HIV infection and primary immunodeficiency may develop NTM pulmonary disease as part of disseminated infections [12].

Diagnosing NTM infections can be challenging, as the symptoms are nonspecific and overlap with other respiratory conditions [13]. Definitive diagnosis involves isolating the NTM from clinical samples and identifying the specific species through laboratory techniques.

Among pathogenic NTM species, *M. triviale* is rare in humans (0.09% of detected NTM) [14]. *M. avium and M. intracellulare* are the most common (80% of all NTM) [15, 16]. Much information has been accumulated for the major organisms, including *M. avium, M. intracellulare, M. kansasii, M. abscessus*, and *Mycobacterium fortuitum.* However, little is known about other rare NTM, including *M. triviale.*

The MABC is a rapidly growing NTM that can cause human diseases. It is widely distributed in various environments and consists of three subspecies: *M. abscessus* subsp. *abscessus* (Mab), *M. abscessus* subsp. *massiliense* (Mma), and *M. abscessus* subsp. *bolletii* (Mbo) [17].

Although the distribution of the MABC is regional, it is the major NTM causing respiratory infections worldwide, accounting for up to 80% [18]. It most commonly occurs in immunocompromised patients, such as those with cystic fibrosis (CF), HIV-positive patients, COPD patients, and bronchiectasis patients [18]. MABC-induced pulmonary infections have become an important global threat to CF patients. They accelerate inflammatory lung damage, leading to increased morbidity and mortality [19].

Due to the diverse nature of NTM species, their varying antibiotic susceptibilities, and the complexity of infections [20], managing NTM infections requires a multidisciplinary approach involving infectious disease specialists, pulmonologists, and other relevant healthcare professionals [21]. Patients with suspected NTM infections must seek medical attention, undergo proper diagnostic testing, and adhere to the prescribed treatment regimen under healthcare professionals’ guidance.

In summary, understanding the distribution of NTM is crucial for clinical treatment decisions. Furthermore, exploring the correlation between phenotypic drug susceptibility and genetic mutations has profound implications for the rapid prediction of drug susceptibility. In essence, research on NTM is critical for addressing the challenges posed by these infections, improving patient outcomes, developing effective public health strategies, and advancing our understanding of the complex interactions between these bacteria and human health. The present study was designed to understand the distribution of NTM in clinically isolated samples in Henan Chest Hospital.

## Methodology

### Ethical approval number

This study was ethically approved by the Ethics Committee of Henan Chest Hospital, China (Henan Chest Hospital-2023). Informed consent was obtained from each patient. However, data had not been linked to individual patients.

### Sample collection

A total of 420 TB-suspect sputum samples were collected from patients referred to the Henan Chest Hospital Laboratory. The patients sought a diagnosis at the Tuberculosis Clinical Research Centre, Henan Province, China. Medical records and routine analysis, including species identification, patient demographic characteristics, gender, age, regimens, and previous history and outcomes, were carried out.

### Sample processing and culturing

All the samples were cultured on Mycobacteria Growth Indicator Tube (MGIT) and Lowenstein-Jensen (LJ). Single colonies were picked from LJ. Culture medium (Baso Diagnostics Inc., Zhuhai, China), inoculated into 7H9 supplemented with Tween-80 (0.05%) and OADC (10%), were placed at 37 °C on a shaking incubator.

### Identification

All NTM isolates were identified using the Kangli Medical Service Mycobacterial Species Identification Array Kit (TBC and NTM nucleic acid mass spectrometry detection (rapid detection + strain identification) (http://www.kanglijianyan.com/Product/Detail/471)) following the manufacturer’s guidelines, and appropriate quality control measures were taken to ensure reliable results. The kit identifies eight MTBC and more than 32 NTM species (including *Mycobacterium chelonae*, *M. kansasii*, and *M. intracellulare* with only one test).

This kit utilizes nucleic acid mass spectrometry, allowing for rapid detection, strain, and identification. Using this single test, clinicians and researchers can accurately identify the presence of MTBC and a wide range of NTM responsible for various human infections.

## Results

### Prevalence of NTM species

A total of 420 samples under suspicion were subjected to screening for NTM and Mtb infections. Out of these, 188 (44.7%) samples were from female, and 232 (55.3%) from male, resulting in a gender distribution of 1:1.2, and the mean age of patients was 41.2 years. Among the 420 samples, 358 (85.2%) were positive, including 285 (67.8%) positive for Mtb infection and 73 (17.3%) positive for NTM (Table 1). Among the NTM infections, 35 (8.3%) were pure infections and 38 (9%) were mixed infections containing both Mtb and NTM. A total of 62 (14.7%) samples were negative, as detailed in Supplementary material S1.

**Table 1.** Summary of 420 TB patients

No	Infection type	Number
1.	TB	285
2.	TB + NTM	38
3.	NTM	35
4.	Negative	62

In the present study, *M. intracellulare* was the most prevalent NTM species (Table 2), followed by *M. abscessus, M. chelonae,* and *M. triviale.* *M. abscessus* was detected in eleven isolates (15%, 11/73): four were mixed samples with Mtb, two were mixed with *M. intracellulare,* and five were only infected with *M. abscessus.*

**Table 2.** Prevalence of *Mycobacterium abscessus* and its mixed infections

No.	Infection/samples	NTM species	Frequency
1.	Mtb and NTM	*M. abscessus*	4
2.	NTM	*M. abscessus*	5
3.	NTM	*M. abscessus/Mycobacterium intracellulare*	2
4.	NTM	*M. intracellulare*	11
5.	Mtb and NTM	*M. intracellulare*	7
6.	NTM	*M. intracellulare/Mycobacterium branderi*	1
7.	Mtb and NTM	*M. intracellulare/Mycobacterium chelonae*	1
8.	Mtb and NTM	*M. triviale*	3
9.	NTM	*M. triviale*	6
10.	Mtb and NTM	*M. chelonae*	8
11.	NTM	*M. chelonae*	1
12.	Mtb and NTM	*M. chelonae/M. intracellulare*	1

*M. intracellulare* was the most prevalent species in Henan Chest Hospital (n = 23/73, 30.1%) (Table 2). This high prevalence of *M. intracellulare* among pure NTM and mixed infections is alarming. Eleven patients were solely infected with *M. intracellulare* (11/73, 15%), one was infected with *Mycobacterium branderi/M. intracellulare*, and one was infected with *M. chelonae/M. intracellulare.* Eight isolates were mixed (8/73, 10.9%) and infected with Mtb/*M. intracellulare*, and one with Mtb*/M. intracellulare/M. chelonae.* One mixed infection of *M. branderi/M. intracellulare* has also been reported for the first time in our study (Table 2).

Despite its name, *M. triviale* has garnered interest in medical and scientific communities due to its potential implications for human health. *M. triviale* was rarely found in TB infections. However, in this study, nine isolates (9/73, 12.3%) were *M. triviale.* Three were mixed infections with Mtb, and the remaining six were purely *M. triviale* (Table 2). The rate of *M. triviale* in pure NTM infections and mixed infections was high in the present study (12.3%).

*M. chelonae* was detected in ten samples (13%, 10/73), including *M. chelonae/M. intracellulare.* Most of these were mixed, including eight mixed infections with Mtb*;* one was Mtb*, M. intracellulare*, and *M. chelonae.* Only one sample was found to be purely *M. chelonae* (Table 2).

Other low-frequency NTM infections are listed in Table 3. Of seventy-three NTM-positive cases, seven (9.5%) samples were infected with *M. gordonae* with Mtb, one solely infected with *M. gordonae* (Table 3), three were *M. flavum* (4.1%), and one each with *M. triplex* and *M. Kansasii.* All these were mixed infections, including Mtb and NTM species. Other rare NTM species are presented in Supplementary material S1.

**Table 3.** Other NTM infections with tuberculosis

No.	Infection	NTM species	Frequency
1.	Mtb and NTM	*M. gordonae*	*6*
2.	Mtb and NTM	*Mycobacterium nonchromogenicum*	*1*
3.	Mtb and NTM	*M. triplex*	*1*
4.	Mtb and NTM	*Mycobacterium kansasii*	*1*
5.	NTM only	*M. flavum*	*2*
6.	NTM only	*M. myxogenes*	*1*
7.	Mtb and NTM	*M. flavum*	*1*
8.	NTM only	*M. gordonae*	*1*

Mtb, *M. tuberculosis.*

## Discussion

Studies on NTM are essential for better public health management as they provide a better understanding of epidemiology to develop effective strategies for preventing, diagnosing, and treating NTM infections, ultimately improving the overall health outcome. Usually, species identification of NTM is not performed in clinical practice, so misdiagnosis and incorrect treatment are common, which may lead to more severe conditions in future and cause a significant economic burden on healthcare systems and individuals.

The present study was conducted for about 18 months (July 2021 to March 2023) to find the prevalence of NTM species in Henan. To our knowledge, it is the first comprehensive study that reports the prevalence of NTM in Henan Province. The distribution of NTM species in China varies across different regions. In Beijing, the MABC was 73.8% (76/103) and 26.2% (27/103), respectively [22]. Wu et al. reported a prevalence of 5.9% for NTM in Shanghai [4], while Gao et al. identified 43 samples (43/200, 21%) as MABC, including 32 (74.4%) Mab and 11 (25.6%) Mma [23]. Similarly, Mab, Mbo, and Mma accounted for 75.2% (97/129), 14.7% (19/129), and 10.1% (13/129) of the MABC isolates, respectively [24]. Compared with this study, significant differences in each NTM species infection were observed in clinical settings. Yu et al. reported a prevalence of 4.0% (160/3995) for NTM infections in southern central China [25]. In a recent study, the prevalence of NTM infections was 6.4% (317/4917) in overall China and 7.7% in southern China [26]. Our study is the first to investigate the distribution of NTM species in Henan Province, China, thus providing additional data for the southern region. However, pulmonary infections due to NTM are increasing globally [27].

The increased prevalence of pulmonary NTM infections is a major health issue in China [28]. In the present study, the most prevalent NTM was *M. intracellulare,* followed by *M. abscessus.* *M. intracellulare* is closely related to *M. avium* and is commonly referred to as MAC along with *M. avium* [27]. *M. intracellulare* is an opportunistic pathogen that can cause a group of MAC infections. Similar to our results, a previous study [29] from Zhejiang Province reported a 6.2% prevalence (24/390) of NTM in TB-positive cases. The most prevalent NTM species was *M. intracellulare* (16/24, 66.7%), followed by *M. abscessus* (3/24). Zhang et al. analysed 452 NTM isolates in which *M. intracellulare* (188/452; 41.6%) was the most prevalent. Species identification is important for effective treatment because *M. intracellulare* is more sensitive to moxifloxacin and linezolid than *M. avium*, which are more susceptible to rifampicin (RIF) [28].

Mixed infections of NTM and Mtb may further complicate the treatment outcomes, which comprised about 2.6% in a previous study [30]. The prevalence rates of mixed infections are different in regions of China, ranging from 0% in Beijing (0/213) to 3.4% (12/353) in Fuzhou and 3.7% in Guangzhou [31]. In our study, 38 (9%) isolates were mixed infections. This high prevalence of mixed infection in our study may further affect the efforts against TB prevention.

Mixed infections involving *M. chelonae* and Mtb are rare but may occur in complex with other NTM [32]. *M. chelonae*, a rapidly growing NTM [33], and Mtb, the causative agent of TB, are distinct species with different clinical characteristics. Diagnostic challenges can arise when these two pathogens coexist in the same host. A successful strategy for handling mixed infections should consider the variations in antimicrobial susceptibility profiles and treatment options for each species involved.

Infections caused by *M. triviale* are rare and infrequently reported in clinical practice. *M. triviale* rarely causes respiratory disease in humans and accounts for a prevalence of only 0.09% of NTM species [34]. However, contrary to the previous reports, the present study found that *M. triviale* was the third most prevalent NTM in Henan Province (Table 3). *M. triviale* is generally considered nonpathogenic [34]. However, there have been isolated cases where *M. triviale* has been associated with opportunistic infections in individuals with compromised immune systems or pre-existing lung conditions [34]. These infections, although uncommon, underscore the importance of understanding the potential interactions between environmental mycobacteria and human health. Due to its minimal virulence and infrequent association with TB, *M. triviale* infections are have not been extensively studied. Investigating the epidemiology and transmission dynamics of *M. triviale* infections can provide insights into effective management strategies and prevalence among diverse populations. Given the high prevalence rate of this rare pathogen, molecular characterization and treatment methods for this bacterium need to be elucidated for better management of NTM in China.

In this study, a single mixed infection of *M. triplex* and Mtb was also detected (Table 3). This pathogen is slow-growing and rarely causes human disease. *M. flavum* has been known to occasionally cause infections in humans, particularly in individuals with compromised immune systems or underlying lung conditions. Some other rare NTM species detected in the present study include *M. gordonae* and *Mycobacterium nonchromogenicum* (Table 3). Previous studies reported that *M. gordonae* causes disease in immunosuppressed and immunocompetent patients [35, 36]. Tsukamura et al., for the first time, reported *M. nonchromogenicum* as a pathogenic NTM, causing pulmonary infections [37]. Later, some other studies also reported the pathogenic nature of *M. nonchromogenicum* [9, 38, 39]. However, *M. nonchromogenicum* infections are very rare and have not been reported in China.

In the present study, we report the first case of *M. nonchromogenicum* and of *M. branderi in* Henan Province, China. These NTM have previously been sporadically isolated from cases involving pulmonary and wound lesions observed in individuals with intact immune systems [40, 41]. However, the present case stands out as a unique instance as it marks the first documented occurrence of this species in an individual with compromised immunity, specifically a patient diagnosed with acute myeloid leukaemia. This observation suggests a broader range of potential *M. branderi* infections than previously recognized, encompassing patients with weakened immune systems.

Studies on NTM clinical issues are limited, including the diverse range of NTM species, limited and heterogeneous clinical data, absence of standardized diagnostic criteria, small sample sizes, geographical variations, selection and publication biases, retrospective designs leading to recall bias, inconsistent antibiotic susceptibility testing methods, incomplete understanding of pathogenicity, difficulties in assessing long-term outcomes, and interactions with host factors. Despite these challenges, these studies are vital for enhancing our comprehension of NTM infections and refining patient management, necessitating careful consideration of these limitations in research design and analysis to ensure robust and applicable outcomes.

## Limitations of the present study

The present study has some limitations. Firstly, the size of the data set is small, and there are no patient sociodemographic data. Additionally, exploration of the transmission network is not within the scope of this research.

## Recommendations

Future research on NTM should focus on expanding data collection with larger sample sizes and incorporating patient sociodemographic information to understand infection dynamics better. Additionally, epidemiological studies investigating drug resistance patterns among NTM species and developing evidence-based treatment guidelines, especially for mixed infections involving Mtb and NTM, may improve patient management. Longitudinal studies tracking infection progression, exploring geographical variations in prevalence, and advanced genomic analysis, along with One Health approaches, may provide insights into NTM evolution, transmission, and sources.

## Conclusion

In the present study, most NTM infections were mixed with Mtb. *M. intracellulare* was the most prevalent NTM, followed by *M. abscessus, M. chelonae,* and *M. triviale* in Henan Chest Hospital. We reported for the first time a high prevalence of respiratory infection caused by a very rare pathogen, *M. triviale.* Some other rare NTM, including *M. gordonae, M. nonchromogenicum,* and *M. triplex,* were also detected. NTM infections are not limited to specific regions and can have global health implications. Understanding the distribution and transmission dynamics of NTM worldwide is crucial for coordinated efforts in surveillance, prevention, and control on an international scale. Mixed infections involving NTM and Mtb represent a complex and intriguing intersection of mycobacterial pathogens. Clinicians must navigate the complexities of balancing treatment protocols and monitoring patients closely to ensure successful outcomes, making multidisciplinary collaboration and individualized care paramount in addressing this unique challenge at the intersection of TB and NTM infections.

## Supporting information

Hongmei et al. supplementary material Hongmei et al. supplementary material

## Supplementary material

For supplementary material accompanying this paper visit http://dx.doi.org/10.1017/S095026882400061X. click here to view supplementary material
